# A New Benzofuran Glucoside from *Ficus Tikoua* Bur

**DOI:** 10.3390/ijms12084946

**Published:** 2011-08-03

**Authors:** Shao-Peng Wei, Jie-Yu Luan, Li-Na Lu, Wen-Jun Wu, Zhi-Qin Ji

**Affiliations:** 1 State Key Laboratory of Crop Stress Biology in Arid Areas, Northwest A & F University, Yangling 712100, Shaanxi, China; E-Mails: weishaopeng8888@163.com (S.-P.W.); wuwenjun@nwsuaf.edu.cn (W.-J.W.); 2 Key Laboratory of Plant Protection Resources and Pest Integrated Management, Ministry of Education, College of Plant Protection, Northwest A & F University, Yangling 712100, Shaanxi, China; 3 Shaanxi Province Key Laboratory Research & Development on Botanical Pesticide, Northwest A & F University, Yangling 712100, Shaanxi, China; E-Mail: meiyadeselang@163.com; 4 Xi’an Modern Chemistry Research Institute, Xi’an 710065, Shaanxi, China; E-Mail: wmc204@yahoo.com.cn

**Keywords:** *Ficus tikoua* Bur., benzofuran glucoside, antioxidant activity

## Abstract

From the water-soluble portion of the methanol extract of stems of *Ficus tikoua* Bur., a new benzofuran glucoside, named 6-carboxyethyl-5-hydroxybenzofuran 5-*O*-β-d-glucopyranoside (**1**), together with one known benzofuran glucoside (**2**) were isolated. Their structures were elucidated by 1D and 2D (^1^H-^1^H COSY, HMQC, and HMBC) NMR spectroscopy and HRMS techniques. The antioxidant activities of the isolated compounds were assayed based on the scavenging activities of DPPH free radical. Compounds **1** and **2** exhibited moderate antioxidant activities, and the IC_50_ values were 242.8 μg·mL^−1^ and 324.9 μg·mL^−1^, respectively.

## 1. Introduction

**Figure 1.**
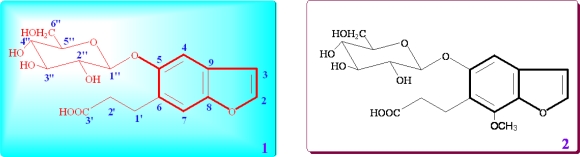
Structures of benzofuran glucosides **1** and **2.**

*Ficus tikoua* Bur., a woody plant of Ficus genus, is widely distributed in south China, India, Vietam and Laos. It has long been used in traditional folk medicine to treat human diseases, such as chronic bronchitis, diarrhea, dysentery, mastadenitis, rheumatism, edema, impetigo, and so on [1,2]. The plants of Ficus genus have attracted considerable attention for pharmacologists due to a wide range of biological properties, including antioxidant [3–5], anti-inflammatory [6–8], cytotoxicity [9,10], antibacterial [6] and antifungal activities [11]. Previous studies show that the phenylpropanoids [12], flavonoids [11], coumarins [2], lignans [4], chromones [13], triterpenoids [14], sesquiterpenoids [15] and alkaloids [16] are the most widespread of the secondary metabolites isolated from the genus Ficus. However, only a few report about the chemical constituents of *F. tikoua* Bur. according to the published literature. As part of our ongoing search for novel secondary metabolites, the constituents of *F. tikoua* Bur. were investigated. This work has led to the isolation of a new (**1**) and one known (**2**) benzofuran glucoside from the water-soluble fraction of the methanol extract of *F. tikoua* Bur. (Figure 1). In this paper, the isolation, structure elucidation and the antioxidant activities of the isolates are presented.

## 2. Results and Discussion

### 2.1. Isolation and Identification

Air-dried stem *F. tikoua* Bur. was extracted with methanol to obtain the crude extract. This residue was suspended in water and partitioned with ethyl acetate. The aqueous phases were subjected to column chromatography (macroporous and anion exchange resin) and reversed-phase HPLC to yield compounds **1** and **2**. Their structures were elucidated on the basis of UV, ESI-MS/MS, HRMS and NMR spectroscopic data.

**Figure 2.**
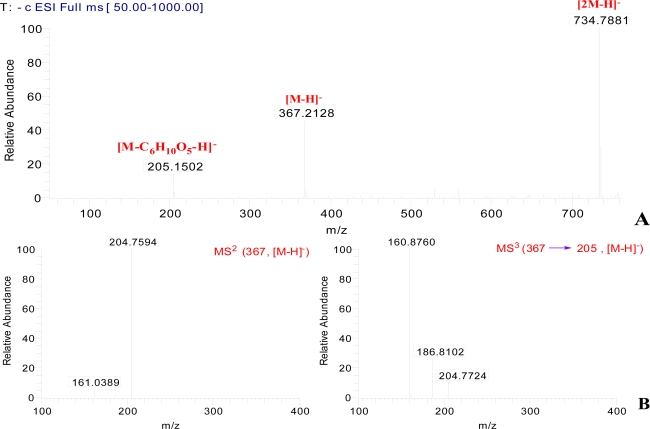
ESI-MS (**A**) and ESI-MS^n^ (**B**) for compound **1** in negative mode.

**Figure 3.**
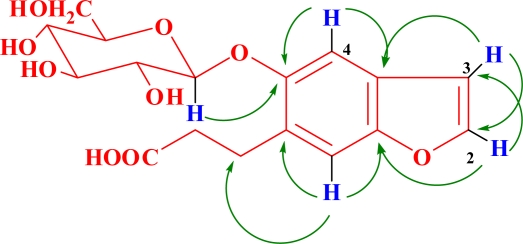
Key HMBC correlations for compound **1**.

**Scheme 1.**
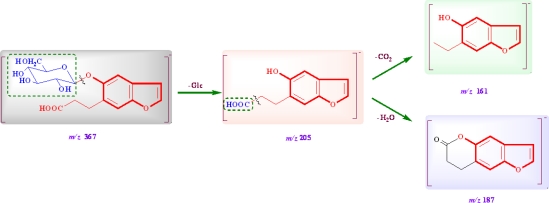
Proposed fragmentation pathways of compound **1**.

Compound **1** (C_17_H_20_O_9_, an amorphous powder, [α]_D_ −29.0°, MeOH), had a molecular ion peak at *m/z* 386.1449 ([M+NH_4_]^+^, calcd. 386.1446) in HR-ESI-MS, in agreement with the molecular formula C_17_H_20_O_9_. ESI-MS showed [2M-H]^−^, [M-H]^−^ and [M-C_6_H_10_O_5_-H]^−^ ion peaks at *m/z* 734.7881, 367.2128 and 205.1502 in the negative mode (Figure 2; Scheme 1). The acid hydrolysis of **1** gave d-glucose as a sugar component. The ^1^H-, ^13^C- and ^13^C-^1^H correlation spectroscopy (COSY) NMR spectral revealed the presence of one tetrasubstituted benzene, one disubstituted double bond, one carboxyethyl and one β-glucopyranosyl unit in **1** (Table 1). The β-configuration of the anomeric center of glucopyranosyl was suggested by the large coupling constant (*J* = 7.5 Hz). Comparison of its NMR data with Glycoside 8 [17,18], which was isolated from the fruit of *Glehnia littoralis*, suggested that **1** also is a glucopyranoside of a benzofuran derivative and might be the regioisomer of the cnidioside A. This assumption was supported by a heteronuclear multiple-bond correlation (HMBC) experiment. The analysis of HMBC spectral and ^1^H-^1^H COSY spectral data (Figure 3) suggested a carboxyethyl group at C-6. The position of attachment of the glucosyl unit was revealed to be C-5 from the H-C long-range correlation between the glucosyl anomeric proton signal and the C-5 carbon in the HMBC spectrum. Therefore, **1** was characterized as 6-carboxyethyl-5-hydroxy-benzofuran 5-*O-*β-d-glucopyranoside.

Compound **2** (C_18_H_22_O_10_) was identified as 6-carboxyethyl-7-methoxyl-5-hydroxy-benzofuran 5-*O*-β-d-glucopyranoside based on UV, ESI-MS, ^1^H- and ^13^C-NMR spectroscopic data (Table 1) [17].

### 2.2. Antioxidant Activity

Further, to assess the possible utilization of isolated compounds, the antioxidant activities were evaluated. DPPH is widely used to evaluate antioxidant capacity and changes color from purple to yellow upon acceptance of electrons/hydrogens, thus indicating scavenging activity. It was observed that compounds **1** and **2** moderately transformed the DPPH radical into its reduced form, and the IC_50_ values were 242.8 and 324.9 μg·mL^−1^, respectively. It was very interesting to note that **2** exhibited weaker activities than **1**, suggesting that the methoxy at C_7_ might has a negative influence on antioxidant activity.

## 3. Experimental Section

### 3.1. General

Solvents were of analytical reagent (AR) grade unless otherwise mentioned. Flash column chromatography (FC): C_18_ silica gel (particle size 15 μm; Fuji Silysia Chemical Ltd.). HPLC: Shimadzu-6AD HPLC apparatus; Hypersil ODS_2_ column (250 × 4.6 mm; 5 μm) and (250 × 20 mm; 10 μm), MeOH: H_2_O = 2:8 as eluent; UV detector at 230 nm. Specific rotations, Perkin-Elmer 341 polarimeter; ^1^H and ^13^C-NMR Spectra: Bruker-Avance-500 spectrometer; MeOD as solvent. HR-ESI-MS: Bruker Apex II mass spectrometer; ESI-MS: Thermo Finnigan LCQ Advantage MAX LC/MS mass spectrometer (USA); molecular scan range 100–1000 amu.

### 3.2. Plant Material

The stem of *F. tikoua* Bur. was collected in Hongya County, Sichuan Province, P.R. China, in September 2009, and authenticated by Prof. Hua Yi (College of Life Sciences, Northwest Agricultural and Forestry University). The voucher specimens (samples No. NWAU2009-FT15) were deposited with the College of Life Sciences, Northwest Agricultural and Forestry University.

### 3.3. Extraction and Isolation

The dried and pulverized stem (5.0 kg) of *F. tikoua* Bur. was extracted with methanol (10 L × 6) under reflux for 4 h. The solvent was evaporated under reduced pressure to give a residue (280.0 g), equivalent to 5.6% of the weight of the dried sample. This residue was suspended in water (5 L) and partitioned with ethyl acetate (5 L × 3). The aqueous phase was subjected to column chromatography (12.0 × 150 cm) packed with 2.0 kg D101 macroporous resin and gradiently eluted with mixed H_2_O and MeOH (100:0, 80:20, 60:40, 40:60, 20:80 and 0:100; 6 L of eluent for each step), 72 fractions of *ca.* 500 mL each which were combined to 6 fractions (HPLC monitoring). Then Fraction 2 was successively loaded on anion exchange resin column chromatography (5.5 × 100 cm, eluted with 2% HCl), reversed-phase flash column (C_18_, MeOH:H_2_O). In this way, after column chromatography (macroporous and anion exchange resin), reversed-phase flash column and pre-HPLC, a new benzofuran glucoside (**1**, 48 mg) and 6-carboxyethyl-7-methoxy-5-hydroxybenzofuran-5-*O*-β-d-glucopyranoside (**2**, 103 mg) were isolated.

### 3.4. Antioxidant Assays

Antioxidant potential of compounds **1** and **2** were determined by a modification of the 1,1-diphenyl-2-picrylhydrazyl (DPPH) radical scavenging method. Free radical scavenging activity of the tested sample against stable DPPH was determined spectrophotometrically by the slightly modified method of Gyamfi [19]. When DPPH reacts with an antioxidant, which can donate hydrogen, it is reduced. The changes in color (from deep-violet to light-yellow) were measured at 517 nm on a UV/vis light spectrophotometer. Fifty microliters of methanol solution of **1** and **2** at concentrations of 0.1, 0.2, 0.5, 1, and 2mg·ml^−1^, respectively, in each reaction was mixed with 1 mL of 0.1 mM DPPH in methanol solution and 450 μL of 50 mM Tris-HCl buffer (pH 7.4). After 30 min of incubation at room temperature the reduction of the DPPH free radical was measured spectrophotometrically. Methanol and tert-butyl hydroquinone (TBHQ) were used as negative and positive control, and all tests were carried out in triplicate. IC_50_ values (concentration of sample required to scavenge 50% of free radicals) were calculated from the regression equation, prepared from the concentration of the tested sample. Percentage inhibition of free radical formation/percentage inhibition DPPH was calculated from the following equation:

$$\text{Inhibition}\%\;=\;\frac{\text{Absorbance of control }-\;\text{Absorbance of test sample}}{\text{Absorbance of control}}\times 100$$

The data were statistically analyzed by using Student’s *t*-test and analysis of variance for individual parameters was performed by Duncan’s test on the basis of mean values to find out the significance at *p* < 0.05. Correlation between antioxidant activities were carried out using the correlation and regression in the EXEL program.

## 4. Conclusions

A new benzofuran glucoside (6-carboxyethyl-5-hydroxybenzofuran 5-*O*-β-d-glucopyranoside), together with one known benzofuran glucoside (6-carboxyethyl-7-methoxyl-5-hydroxy-benzofuran 5-*O*-β-d-glucopyranoside) were isolated from the water-soluble portion of *Ficus tikoua* Bur. The IC_50_ values were 242.8 μg·mL^−1^ and 324.9 μg·mL^−1^, based on the scavenging activities of DPPH free radical, respectively. It is suggested that *Ficus tikoua* could be considered as a source of antioxidant agent which might be applied in pharmaceutical and cosmetic products.

## Figures and Tables

**Table 1.** The ^1^H and ^13^C-NMR chemical shifts of compound **1** (D_2_O, 500 MHz).

**No.**	**1**		**2**	

	***δ*_C_**	***δ*_H_ (*J*/Hz)**	***δ*_C_**	***δ*_H_ (*J*/Hz)**
C-2	145.91 d	7.61 d (2.0)	144.65 d	7.78 d (2.0)
C-3	107.15 d	6.70 d (2.0)	104.53 d	7.10 d (2.0)
C-4	99.77 s	7.34 s	94.33 s	7.26 s
C-5	155.01 s		153.45 s	
C-6	127.66 s		116.15 s	
C-7	122.13 s	7.37 s	150.83 s	
C-7(OMe)			60.93 q	4.16 s
C-8	155.80 s		155.19 s	
C-9	123.23 s		113.75	
C-1′	27.75 m	2.99∼3.08 m	19.46 t	3.18 t (7.5)
C-2′	36.61 m	2.61∼2.64 m	34.70 t	2.71 t (7.5)
C-3′	178.84 s		178.87 s	
Glc				
1″	103.22 d	4.93d (7.5)	101.26 d	5.20 d (7.5)
2″	75.00 d	3.69 m	73.29 d	3.89 m
3″	78.18 d	3.62 m	76.10 d	3.78 m
4″	71.40 d	3.70 m	69.75 d	3.76 m
5″	78.18 d	3.29 m	76.37 d	3.39 m
6″	62.56 t	3.80, 4.02 m	60.72 t	3.67, 4.08 m
